# Protease inhibitor-induced nausea and vomiting is attenuated by a peripherally acting, opioid-receptor antagonist in a rat model

**DOI:** 10.1186/1742-6405-6-19

**Published:** 2009-08-21

**Authors:** Chun-Su Yuan, Chong-Zhi Wang, Sangeeta R Mehendale, Han H Aung, Adela Foo, Robert J Israel

**Affiliations:** 1 Department of Anesthesia & Critical Care, University of Chicago, Chicago, USA; 2 Committee on Clinical Pharmacology and Pharmacogenomics, Pritzker School of Medicine, University of Chicago, Chicago, USA; 3 Progenics Pharmaceuticals Inc, Tarrytown, NY, USA

## Abstract

**Background:**

Protease inhibitors such as ritonavir can cause nausea and vomiting which is the most common reason for discontinuation. Rats react to nauseous and emetic stimuli by increasing their oral intake of non-nutritive substances like kaolin, known as pica behavior. In this study, we evaluated the effects of methylnaltrexone, a peripherally acting *mu*-opioid receptor antagonist that does not affect analgesia, on ritonavir-induced nausea and vomiting in a rat pica model.

**Results:**

We observed that 24 to 48 hr after administration of oral ritonavir 20 mg/kg, kaolin consumption increased significantly in rats (*P *< 0.01). This increase was attenuated by pretreatment with an intraperitoneal injection of methylnaltrexone (0.3–3.0 mg/kg) in a dose dependent manner (*P *< 0.01) and also with naloxone (0.1–0.3 mg/kg) (*P *< 0.01). The areas under the curve for kaolin intake from time 0 to 120 hr were significantly reduced after administration of the opioid antagonists. Food intake was not significantly affected. Plasma naltrexone levels were measured after methylnaltrexone injection, and no detectable levels were found, indicating that methylnaltrexone was not demethylated in our experimental paradigm.

**Conclusion:**

These results suggest that methylnaltrexone may have potential clinical utility in reducing nausea and vomiting in HIV patients who take ritonavir.

## Introduction

Infection with the human immunodeficiency virus (HIV), which may progress to acquired immune deficiency syndrome (AIDS), is a deadly disease that affects many millions of people worldwide [1,2]. If patients are not treated in a timely fashion, the disease can cause morbidity and lead to death because of immune dysfunction and opportunistic infections. To reduce viral loads and improve life expectancy, treatment guidelines require that patients comply with drug regimens for an extended period of time [3,4]. The main obstacles to such compliance are treatment-induced adverse effects. Adverse effects not only deteriorate quality of life, but negatively affect compliance [5]. Nausea and vomiting are examples of drug-induced adverse effects that may affect compliance [4,6,7].

Protease inhibitors are commonly used potent anti-HIV drugs. Drugs in this class, especially ritonavir, induce nausea and vomiting [8]. Ritonavir is used in anti-HIV therapy as an adjuvant to other protease inhibitors because it inhibits the hepatic CYP 3A enzyme, thereby increasing the bioavailability and plasma concentration of other antiviral agents [9,10]. Although the dose required for the adjuvant effects of ritonavir is lower than that required for its direct antiviral effect, nausea and emesis have been reported in at least 20% of the patients taking it [4].

In rats, emetic stimuli alter feeding habits, manifested as pica behavior, i.e., an increased consumption of non-nutritive substances such as kaolin, a type of clay [11-13]. Using the rat and pica model, we previously quantified kaolin consumption as a measure of nausea and vomiting. We observed that drug-induced consumption of pica was decreased by administration of selected pharmacological agents [14-16].

**Figure 1:**
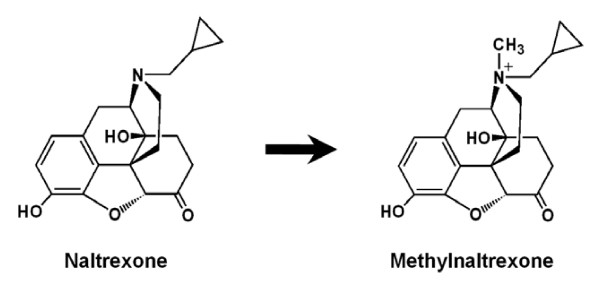
**Chemical structures of naltrexone and methylnaltrexone**.

Methylnaltrexone is a novel peripherally acting *mu*-opioid receptor antagonist derived from naltrexone [17] (Fig. 1). In a previous pilot study in healthy subjects, we observed that methylnaltrexone decreased certain opioid-induced troublesome subjective effects, including nausea [18]. In other studies using the rat pica model, methylnaltrexone reduced opioid-induced nausea and vomiting [16]. Although the mechanism by which ritonavir causes nausea and vomiting is unknown, combinations of anti-emetics may partially abate the symptoms of ritonavir [19,20]. In this study, we evaluated the effects of methylnaltrexone on ritonavir-induced nausea and vomiting in the rat pica model. Naloxone, a non-selective opioid receptor antagonist, was also used for comparison with the methylnaltrexone for effect and site of action.

## Methods

### Animals

The experimental protocols were approved by the Institutional Animal Care and Use Committee or IACUC of the University of Chicago. Male Wistar strain rats (Harlan Sprague Dawley, Indianapolis, IN), weighing between 150–300 g, were housed in environmentally controlled conditions with a 12 hr light, 12 hr dark cycle. Rats were allowed free access to water and standard laboratory rat chow (Harlan-Teklad, Madison, WI).

### Measurement of pica (kaolin intake)

Kaolin pellets were prepared based on a method described previously [15]. Briefly, pharmacological grade kaolin (or hydrated aluminum silicate; Fisher, Fair Lawn, NJ) and acacia (or gum arabic; Fisher, Fair Lawn, NJ) were mixed using a 99:1 ratio in distilled water. The kaolin paste was rolled and cut into pieces similar in shape to rat chow pellets. The pellets were dried at room temperature for 72 hr.

Rats were placed in individual isolation cages (45 cm × 35 cm × 25 cm) and were allowed access to regular food and kaolin during a 3-day adaptation period before the study period. There were 6–7 rats in each of the four groups: vehicle (saline) plus vehicle, vehicle plus ritonavir, naloxone plus ritonavir, and methylnaltrexone plus ritonavir. Rats received ritonavir 20 mg/kg (Abbott Laboratories, North Chicago, IL) orally via a gavage tube in the morning on 2 consecutive days (0 hr and 24 hr) [21-23]. Vehicle, naloxone 0.1 mg/kg or 0.3 mg/kg (Sigma, St. Louis, MO), or methylnaltrexone 0.3 mg/kg, 1.0 mg/kg, or 3.0 mg/kg (Mallinckrodt Chemicals, St. Louis, MO) pretreatment was administered intraperitoneally [15], 30 min before 20 mg/kg ritonavir administration. Rats were observed immediately, at 15 min, 2 hr, and daily thereafter for signs of distress.

Kaolin and food pellets were weighed to the nearest 0.1 g and placed in containers within the cage each morning. The kaolin and food remaining from the previous day were carefully collected, dried for 72 hr and weighed. Daily kaolin intake and food intake were measured for 5 days following the first ritonavir treatment.

### Blood sample collection

In some experiments, blood samples were collected for the measurement of plasma naltrexone level, an indicator of possible demethylation of methylnaltrexone [24]. The rat was restrained and the tail vein was exposed. The tail was dipped in warm water to help dilate the vessel. A small rubber band was placed around the base of the tail. Blood samples were collected using a microhematocrit tube inserted into the hub of a small needle that was placed into the vein. Blood samples were collected at 0, 10, 20, 30, 60, 90, or 120 min after the first dose of methylnaltrexone.

### Measurement of naltrexone and methylnaltrexone concentrations

Plasma naltrexone and methylnaltrexone levels were determined by high performance liquid chromatography (HPLC) adapted from a previously reported method [25]. Naltrexone and methylnaltrexone were separated from plasma by the solid phase extraction (SPE) technique. Plasma samples (100 to 200 μl) diluted in water were passed through SPE columns (Varian CBA columns, 100 mg, Harbor City, CA) that had been conditioned by n-propanol and water. The analytes were eluted from the columns by the mixture of n-propanol and trifluoroacetic acid (25 mM) in an aqueous solution prepared in 2:1 proportion. The eluate was evaporated to dryness in a stream of nitrogen at 55°C. The residue was reconstituted in the mobile phase, filtered through the nylon HPLC syringe filter and subjected to analysis. In HPLC analysis, an electrochemical detector has high sensitivity for automated, analytical chromatography of electroactive compounds. The HPLC system (Shimadzu Corporation, Kyoto, Japan) and electrochemical detector (ESA Coulochem, model 5100A, Chelmsford, MA) consisted of an LC-10AD pump, SCL-10A system controller, and SIL-10A auto injector equipped with sample cooler. The electrochemical detector worked at the following settings: detector 1, +360 mV, detector 2, +600 mV, guard cell +650 mV. Data were collected using EZChrom 2-2 HPLC software. In the mobile phase we used sodium phosphate 30 mM, sodium acetate 20 mM, methanol 6%, tetrahydrofuran 1% at pH 4.2. The system was calibrated daily in the range of 5 to 100 ng/mL. The practical limit of detection for plasma samples was approximately 2 ng/mL (100 pg/injection).

### Statistical analysis

Data were analyzed with a two-way analysis of variance (ANOVA) with group and time as the two factors. Statistical significance was considered at *P *< 0.05.

## Results

### Effects of naloxone on ritonavir-induced nausea and vomiting

**Figure 2:**
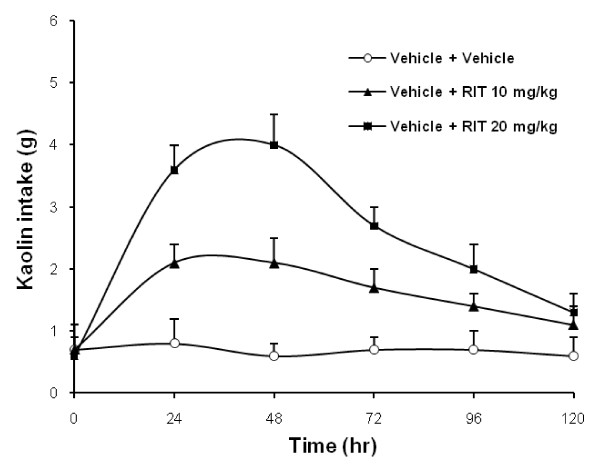
**Dose-related effects of pretreatment with ritonavir on kaolin intake**. Rats treated with saline only consumed < 1.0 g/day of kaolin during 5 consecutive days (0, 24, 48, 72, 96, and 120 hr). Ritonavir doses at 10 and 20 mg/kg significantly increased kaolin consumption at 24 to 48 hr in a dose-related manner (*P *< 0.01). Data are presented as mean ± SEM. n = 6 per group.

In rats treated with saline, less than 1.0 g kaolin was consumed daily during 5 consecutive days. After oral ritonavir doses of 10 and 20 mg/kg, kaolin consumption increased significantly at 24 to 48 hr in a dose-related manner (Fig. 2; *P *< 0.01 comparing the area under the curve or AUC). At 30 mg/kg, kaolin intake did not increase further, and thus, the ritonavir dose used for this study was 20 mg/kg.

**Figure 3:**
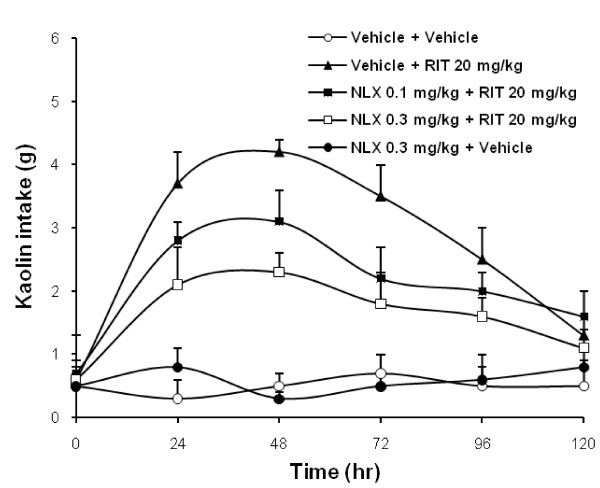
**Effects of pretreatment with naloxone on kaolin intake induced by ritonavir in rats**. Ritonavir-induced increase in kaolin intake was attenuated with naloxone in a dose-related manner (*P *< 0.01). Data are presented as mean ± SEM. n = 6 per group. NLX, naloxone; RIT, ritonavir.

**Figure 4:**
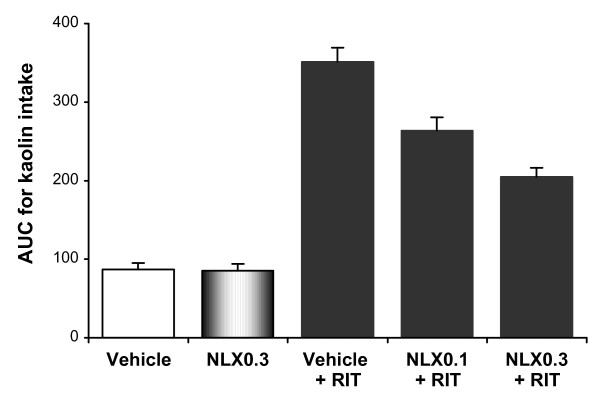
**Area under the curve (AUC) for kaolin intake from time 0 to 120 hr**. Naloxone significantly reduced kaolin intake induced by ritonavir (*P *< 0.01 compared to vehicle). Naloxone 0.3 mg/kg alone did not affect kaolin intake. Data are presented as mean ± SEM. RIT, ritonavir 20 mg/kg; NLX0.1, naloxone 0.1 mg/kg; NLX0.3, naloxone 0.3 mg/kg.

Fig. 3 shows that the ritonavir-induced increase in kaolin intake was attenuated by 0.1 or 0.3 mg/kg pretreatment with naloxone. The AUC for kaolin intake from time 0 to 120 hr, vehicle plus vehicle was 87 ± 8.1 g•hr, naloxone 0.3 mg/kg plus vehicle was 86 ± 9.2 g•hr, vehicle plus ritonavir was 351 ± 18.2 g•hr, naloxone 0.1 mg/kg plus ritonavir was 264 ± 16.7 g•hr, and naloxone 0.3 mg/kg plus ritonavir was 205 ± 11.3 g•hr (Fig. 4; *P *< 0.01).

With naloxone 0.3 mg/kg alone, kaolin intake was not significantly affected (Fig. 4). In all test groups, food intake was not significantly affected.

### Effects of methylnaltrexone on ritonavir-induced nausea and vomiting

**Figure 5:**
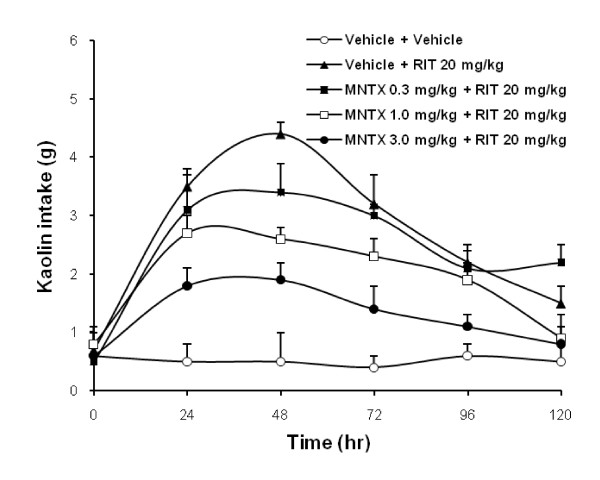
**Effects of pretreatment with methylnaltrexone on kaolin intake induced by ritonavir in rats**. Ritonavir-induced increase in kaolin intake was attenuated with methylnaltrexone in a dose-related manner (*P *< 0.01). Data are presented as mean ± SEM. n = 6–7 per group. MNTX, methylnaltrexone; RIT, ritonavir.

**Figure 6:**
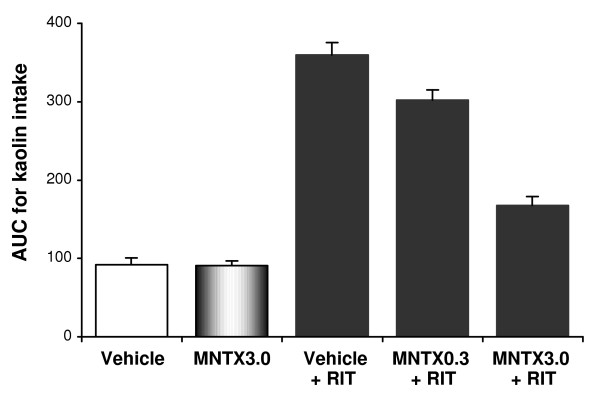
**Area under the curve (AUC) for kaolin intake from time 0 to 120 hr**. Methylnaltrexone significantly reduced kaolin intake induced by ritonavir (*P *< 0.01 compared to vehicle). Data are presented as mean ± SEM. RIT, ritonavir 20 mg/kg; MNTX0.3, methylnaltrexone 0.3 mg/kg; MNTX3.0, methylnaltrexone 3.0 mg/kg.

Effects of pretreatment with methylnaltrexone on kaolin intake after ritonavir are shown in Fig. 5. Kaolin intake induced was attenuated by methylnaltrexone in a dose-dependent manner. The AUC for kaolin intake from time 0 to 120 hr, vehicle plus vehicle was 92 ± 8.6 g•hr, vehicle plus ritonavir was 360 ± 15.7 g•hr, methylnaltrexone 0.3 mg/kg plus ritonavir was 302 ± 13.2 g•hr, methylnaltrexone 1.0 mg/kg plus ritonavir was 242 ± 14.9 g•hr, and methylnaltrexone 3.0 mg/kg plus ritonavir was 168 ± 11.5 g•hr (Fig. 6; *P *< 0.01).

With methylnaltrexone 3.0 mg/kg alone, kaolin intake was not significantly affected (Fig. 6). In all test groups, food intake was not significantly affected.

### HPLC analysis of naltrexone

**Figure 7:**
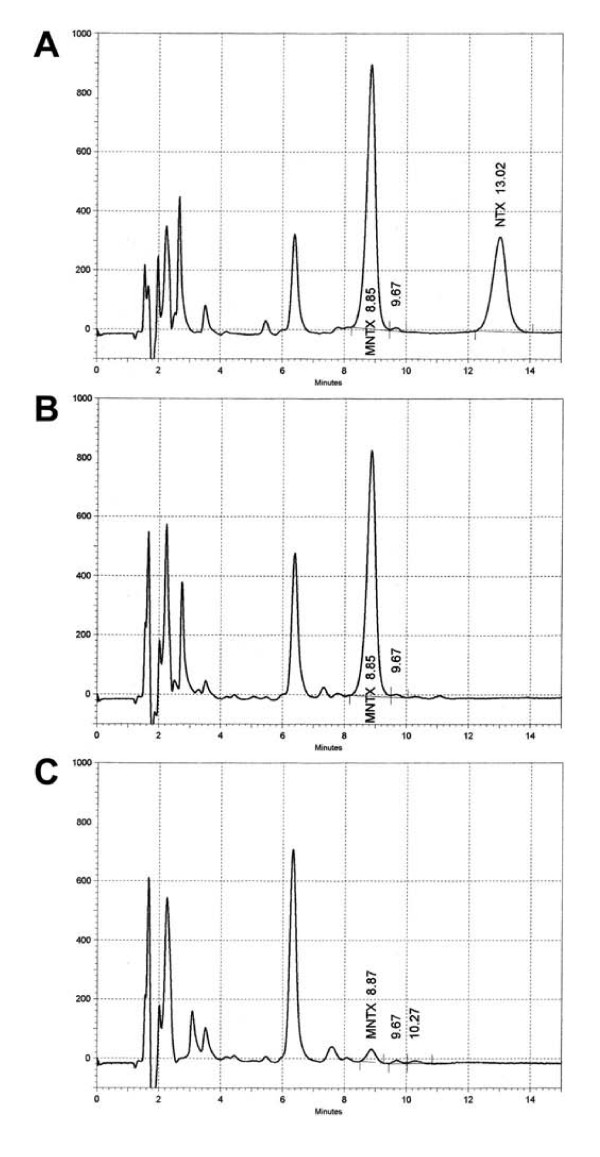
**Representative HPLC chromatograms of methylnaltrexone and naltrexone in plasma samples**. (A), a chromatogram of a standard plasma extract of methylnaltrexone (100.0 ng/mL) and naltrexone (50.0 ng/mL); (B), 92.5 ng/mL methylnaltrexone was detected after 3.0 mg/kg administration; (C), a gradually reduced methylnaltrexone level (4.7 ng/mL) was detected as time elapsed. At all measured time points, no naltrexone level was detected, as shown in (B) and (C). MNTX, methylnaltrexone; NTX, naltrexone.

No detectable naltrexone level was found in association with methylnaltrexone 3.0 mg/kg. In contrast, methylnaltrexone levels were detected after it was administered. Representative chromatograms are shown in Fig. 7.

## Discussion

Protease inhibitors are efficacious antiretroviral agents that produce several adverse effects, especially nausea and vomiting. To date, the mechanism by which protease inhibitors cause nausea or vomiting has not been investigated. Considering that compliance with treatment is a pre-requisite for effective antiviral therapy in patients with AIDS, drug-induced adverse effects that inhibit compliance should be treated. Treatment with conventional anti-emetics, usually in combination, can partially decrease ritonavir-induced nausea and vomiting. Ondansetron, a 5-HT_3 _antagonist, has been used in refractory cases of nausea and vomiting in AIDS patients, in combination with other anti-emetics [14,19,20]. Whether endogenous opioids contribute to the adverse effects in the gut is unknown. In this study, we evaluated the effects of opioid receptor antagonists on ritonavir-induced nausea and vomiting. Naloxone reduced ritonavir-induced nausea and vomiting. A non-selective opioid antagonist such as naloxone, however, compromises opioid analgesia [17]. Therefore, treating ritonavir-induced emesis with naloxone may not be clinically applicable to patients who take opioid medication for AIDS-related pain, which can result from the virus itself, various forms of treatment, opportunistic infections and cancers [26].

Methylnaltrexone (or N-methylnaltrexone bromide) is a quaternary derivative of opioid antagonist, naltrexone (Fig. 1). As tertiary compounds, antagonists such as naloxone, naltrexone and nalmaphene are fairly lipid soluble and cross the blood-brain barrier easily. Addition of the methyl group to naltrexone forms a compound with greater polarity and lower lipid solubility. Thus, methylnaltrexone has restricted access to the blood-brain barrier and decreases the constipating effects of adverse effects of opioid pain medications. Because these effects are mediated by peripherally located receptors, the analgesic effects, which are mediated at receptors in the central nervous system, are spared [27]. In this study methylnaltrexone significantly attenuated non-opioid, protease inhibitor-induced nausea and vomiting. Methylnaltrexone may have clinical value in treating protease inhibitor-induced emesis without affecting analgesia.

A rat pica model was used to evaluate the symptoms of nausea and emesis. Rats exposed to a variety of emetic stimuli feed on non-nutritive substances like clay, a phenomenon called pica behavior. Pica in rats is thus analogous to nausea and vomiting in humans and other species [11,13]. Pica in rats is mediated by mechanisms and receptors involving serotonin and dopamine, similar to those in humans and other species [12,13]. The model has been used extensively and validated in several studies researching anti-emetic drugs [12,16]. A dose-dependent pica response induced by ritonavir has already been demonstrated [14]. In this study, we used the pica model to confirm that treatment with methylnaltrexone significantly reduced ritonavir-induced pica.

In rodents, methylnaltrexone may be partially metabolized via demethylation as measured by the exhaled ^14^CO_2 _breath test [24]. However, systemic methylnaltrexone administration did not antagonize morphine-induced analgesia in rats subjected to the radiant-heat tail-flick assay, and morphine-induced reduction in gastrointestinal tract movement was antagonized by the compound in a dose-related manner [28]. In our study, we used HPLC to measure plasma naltrexone levels after methylnaltrexone administration and no detectable naltrexone level was found with the highest methylnaltrexone dose. In a separated analytical study, a more sensitive LC/MS/MS assay was also used to detect any possible demethylation of methylnaltrexone for up to 6 hr after the first dose of the compound, and results showed that levels of naltrexone were below the limit of detection (unpublished data), suggesting that naltrexone did not play a pharmacodynamic role in our experimental paradigm.

Pain is a major issue for people living with HIV and AIDS, and opioids are widely prescribed for non-cancer and cancer pain conditions [29,30]. In this study, our data suggest that opioid receptor antagonists contribute to relieving protease inhibitor-induced gastrointestinal adverse effects, and thus, methylnaltrexone may have a clinical utility in reducing nausea and vomiting in AIDS patients who take ritonavir. In addition, besides the compound's anti-emetic activity in HIV therapy, methylnaltrexone is also effective in counteracting opioid-induced bowel dysfunction in these AIDS patients without interfering with pain control.

## Competing interests

Methylnaltrexone was originally formulated and subsequently modified by faculty at the University of Chicago. It is currently being developed and commercialized by Progenics Pharmaceuticals and Wyeth Pharmaceuticals, for which CSY serves as a consultant. The University of Chicago and CSY stand to benefit financially from the development of methylnaltrexone. RJI is an employee of Progenics Pharmaceuticals, which has a propriety commercial interest in methylnaltrexone.

## Authors' contributions

CSY was responsible for the study design, collection and assembly of data, analysis and interpretation of the data, drafting of the article, critical revision of the article, final approval of the article, and obtaining of funding. CZW was responsible for the study design, collection and assembly of data, analysis and interpretation of the data, critical revision of the article, and final approval of the article. SRM was responsible for the analysis and interpretation of the data, and critical revision of the article. HHA was responsible for the collection and assembly of data, and analysis and interpretation of the data. AF was responsible for the collection and assembly of data, and drafting of the article. RJI was responsible for the study design, critical revision of the article, and final approval of the article.

## References

[B1] Sande M, Volberding PA (1999). The Medical Management of AIDS.

[B2] Selgelid MJ (2005). Ethics and infectious disease. Bioethics.

[B3] Proctor VE, Tesfa A, Tompkins DC (1999). Barriers to adherence to highly active antiretroviral therapy as expressed by people living with HIV/AIDS. AIDS Patient Care STDS.

[B4] Barlett JG (2004). HIV: Current diagnosis, management, and treatment options. PDR.

[B5] Carr A (2002). Improvement of the study, analysis, and reporting of adverse events associated with antiretroviral therapy. Lancet.

[B6] Lichterfeld M, Nischalke HD, Bergmann F, Wiesel W, Rieke A, Theisen A, Fätkenheuer G, Oette M, Carls H, Fenske S, Nadler M, Knechten H, Wasmuth JC, Rockstroh JK (2002). Long-term efficacy and safety of ritonavir/indinavir at 400/400 mg twice a day in combination with two nucleoside reverse transcriptase inhibitors as first line antiretroviral therapy. HIV Med.

[B7] Gartland M (2001). AVANTI 3: a randomized, double-blind trial to compare the efficacy and safety of lamivudine plus zidovudine versus lamivudine plus zidovudine plus nelfinavir in HIV-1-infected antiretroviral-naive patients. Antivir Ther.

[B8] Elperin A, Sax P (1996). A patient's guide to protease inhibitors. AIDS Clin Care.

[B9] Ernest CS, Hall SD, Jones DR (2005). Mechanism-based inactivation of CYP3A by HIV protease inhibitors. J Pharmacol Exp Ther.

[B10] Motwani B, Khayr W (2006). Pharmacoenhancement of protease inhibitors. Am J Ther.

[B11] Mitchell D, Wells C, Hoch N, Lind K, Woods SC, Mitchell LK (1976). Poison induced pica in rats. Physiol Behav.

[B12] Takeda N, Hasegawa S, Morita M, Horii A, Uno A, Yamatodani A, Matsunaga T (1995). Neuropharmacological mechanisms of emesis. I. Effects of antiemetic drugs on motion- and apomorphine-induced pica in rats. Methods Find Exp Clin Pharmacol.

[B13] Takeda N, Hasegawa S, Morita M, Matsunaga T (1993). Pica in rats is analogous to emesis: an animal model in emesis research. Pharmacol Biochem Behav.

[B14] Aung H, Mehendale S, Chang WT, Wang CZ, Xie JT, Yuan CS (2005). Scutellaria baicalensis decreases ritonavir-induced nausea. AIDS Res Ther.

[B15] Aung HH, Dey L, Mehendale S, Xie JT, Wu JA, Yuan CS (2003). Scutellaria baicalensis extract decreases cisplatin-induced pica in rats. Cancer Chemother Pharmacol.

[B16] Aung HH, Mehendale SR, Xie JT, Moss J, Yuan CS (2004). Methylnaltrexone prevents morphine-induced kaolin intake in the rat. Life Sci.

[B17] Yuan CS, Israel RJ (2006). Methylnaltrexone, a novel peripheral opioid receptor antagonist for the treatment of opioid side effects. Expert Opin Investig Drugs.

[B18] Yuan CS, Foss JF, O'Connor M, Osinski J, Roizen MF, Moss J (1998). Efficacy of orally administered methylnaltrexone in decreasing subjective effects after intravenous morphine. Drug Alcohol Depend.

[B19] Meyer M (1999). Palliative care and AIDS: 2 – Gastrointestinal symptoms. Int J STD AIDS.

[B20] Padua CA, Cesar CC, Bonolo PF, Acurcio FA, Guimaraes MD (2006). High incidence of adverse reactions to initial antiretroviral therapy in Brazil. Braz J Med Biol Res.

[B21] Denissen JF, Grabowski BA, Johnson MK, Buko AM, Kempf DJ, Thomas SB, Surber BW (1997). Metabolism and disposition of the HIV-1 protease inhibitor ritonavir (ABT-538) in rats, dogs, and humans. Drug Metab Dispos.

[B22] Yamaji H, Matsumura Y, Yoshikawa Y, Takada K (1999). Pharmacokinetic interactions between HIV-protease inhibitors in rats. Biopharm Drug Dispos.

[B23] Shibata N, Gao W, Okamoto H, Kishida T, Iwasaki K, Yoshikawa Y, Takada K (2002). Drug interactions between HIV protease inhibitors based on physiologically-based pharmacokinetic model. J Pharm Sci.

[B24] Kotake AN, Kuwahara SK, Burton E, McCoy CE, Goldberg LI (1989). Variations in demethylation of N-methylnaltrexone in mice, rats, dogs, and humans. Xenobiotica.

[B25] Osinski J, Wang A, Wu JA, Foss JF, Yuan CS (2002). Determination of methylnaltrexone in clinical samples by solid-phase extraction and high-performance liquid chromatography for a pharmacokinetics study. J Chromatogr B Analyt Technol Biomed Life Sci.

[B26] Frich LM, Borgbjerg FM (2000). Pain and pain treatment in AIDS patients: a longitudinal study. J Pain Symptom Manage.

[B27] Yuan CS (2007). Methylnaltrexone mechanisms of action and effects on opioid bowel dysfunction and other opioid adverse effects. Ann Pharmacother.

[B28] Gmerek DE, Cowan A, Woods JH (1986). Independent central and peripheral mediation of morphine-induced inhibition of gastrointestinal transit in rats. J Pharmacol Exp Ther.

[B29] Breitbart W, Dibiase L (2002). Current perspectives on pain in AIDS. Oncology.

[B30] Sullivan MD, Edlund MJ, Fan MY, Devries A, Brennan Braden J, Martin BC (2008). Trends in use of opioids for non-cancer pain conditions 2000–2005 in commercial and Medicaid insurance plans: the TROUP study. Pain.

